# Understanding and Managing Dental Fear and Anxiety in Children: An Evidence-Based Clinical Pathway Across Paediatric Dentistry and Child Psychiatry

**DOI:** 10.1192/j.eurpsy.2026.10651

**Published:** 2026-07-31

**Authors:** S. G. Abbas, M. Rashid, H. Tahseen

**Affiliations:** 1 Sixth Form, The Grange School, Northwich; 2 Dentistry, The Woodlands Dental Practice, Middlewich; 3 Psychiatry, Somerset NHS Trust, Taunton, United Kingdom

## Abstract

**Introduction:**

Child dental fear is a common, modifiable barrier to care that predicts avoidance, emergency-driven attendance and poorer oral/psychosocial outcomes in later life. Fear arises through **learned and neurobiological (amygdala–HPA) pathways**. producing heightened autonomic arousal in the dental setting. Embedding early identification and brief interventions in routine dentistry could change trajectories.

**Objectives:**

1. Synthesize evidence on the development and maintenance of dental fear in children;
2. appraise subjective (psychometric/observational) vs objective (physiological) assessment for clinical usability;
3. evaluate behavioural/psychological interventions with real-world feasibility;
4. propose a pragmatic, stepped-care pathway linking paediatric dentistry with child mental health.

**Methods:**

Critical narrative review across paediatric dentistry, clinical psychology and neurobiology. Inclusion prioritised child-focused studies, validated measures (CFSS-DS, DFSS-SF, DAS, Venham), and interventions (Tell-Show-Do, graded exposure, distraction/VR, CBT-informed approaches; pharmacological adjuncts for severe cases). Each source was logged for credibility, strengths/limits, applicability and convergence/divergence; findings were thematically synthesised (aetiology, measurement, management).

**Results:**

*Aetiology:* Fear typically begins in childhood via conditioning, modelling (parental anxiety) and information pathways; neurobiology (amygdala/HPA) explains autonomic hyperarousal and avoidance.

*Measurement:* Subjective tools (CFSS-DS/DFSS-SF) are reliable and chairside-feasible; DAS is less age-specific; observation (Venham) supports non-verbal cases. Objective indices (HR, skin conductance, salivary cortisol) track arousal but are resource-heavy—best for research/specialist clinics. Combining brief subjective screening with selective physiological monitoring optimises validity and practicality.

*Interventions:* Behavioural methods (Tell-Show-Do, graded exposure, distraction/modelling) consistently reduce fear; dentist-communication micro-skills (predictability, choice, control) are key “active ingredients.” CBT-informed packages and VR are promising; pharmacological support is reserved for severe treatment-blocking anxiety.

*Pathway:* (i) Screen (DFSS-SF/CFSS-DS); (ii) Match to stepped care (universal communication+behavioural strategies → CBT-informed/VR → specialist/adjunctive pharmacology); (iii) Review to prevent avoidance cycles.

**Conclusions:**

**Child dental fear is identifiable and treatable.** Embedding screening and stepped behavioural care in routine dentistry, with clear escalation routes, provides a practical bridge to child mental health. Next steps: implement in primary care, test cost-effectiveness of CBT-informed/VR options, and develop low-cost biosensors. This model can reduce avoidance and improve long-term oral-health trajectories.

**Disclosure of Interest:**

None Declared

